# Parliament and the Professions

**Published:** 1920-07-17

**Authors:** 


### Parliament and the Professions.

### Duplicate Artificial Limbs.

A QUESTION to the Ministry of Pensions elicited the information that a duplicate artificial leg has been supplied in 6,362 cases. The number still to be supplied is 20,350.

### Incidence of Tetanus During the War.

MR. CHURCHILL informed Mr. Waterson that complete statistics as to the incidence of tetanus in all theatres of the late war are not yet available. For the period from August 1914 to December 1918 the number of cases of, and deaths from, tetanus reported in France, Belgium, and the United Kingdom among the troops wounded in France and Belgium give approximately an attack rate of 1.22 per 1,000, and a death-rate of .49 per 1,000. No figures are available as to the incidence elsewhere, but Mr. Churchill understood that very few cases had occurred in other theatres.

In answer to a previous question, Mr. Churchill stated that there were six cases of tetanus among other ranks wounded or injured in action during the South African war, or an attack rate of .28 per 1,000 and three deaths; or a death-rate of .14 per 1,000. There were no cases of tetanus among officers.

